# Supplementary material for: Study of the Relationship between Pulmonary Artery Pressure and Heart Valve Vibration Sound Based on Mock Loop
Source: Bioengineering (Basel). 2023 Aug 20;10(8):985. doi: 10.3390/bioengineering10080985 (PMC10451642; doi:10.3390/bioengineering10080985)
Supplement: Supplementary file 1 [file bioengineering-10-00985-s001.zip › bioengineering-2548545-supplementary.pdf]

supplementary materials

Figure S1

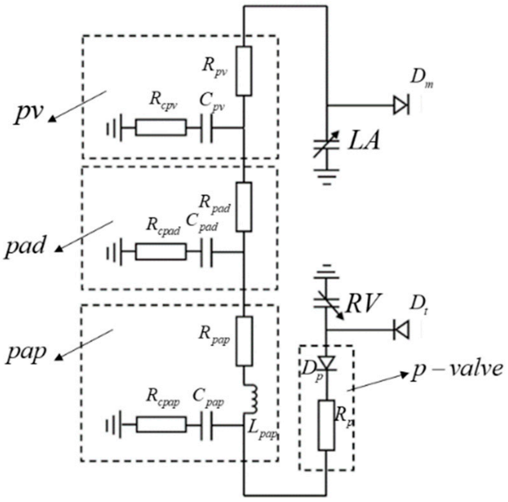

Figure S1 Lumped-parameter circuit platform for pulmonary circulation system. *R* resistances; *C* compliances; *L* inductances; *D* valves; *LA* left atrium; *RV* right ventricle. Full name for the abbreviations used in subscripts: D_m mitral valve; D_t tricuspid valve; D_p pulmonary valve; *pap* proximal pulmonary artery; *pad* distal pulmonary artery; *pv* pulmonary veins; *p*-valve pulmonary valve

Figure S2

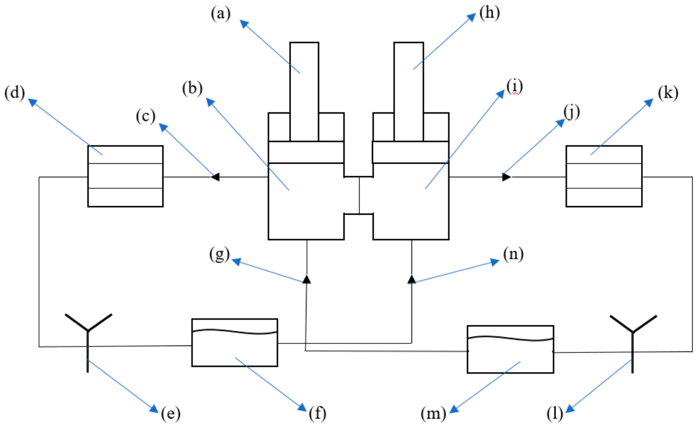

Figure S2 concept diagram of the MCL platform. (a) and (h) servo motor. (b) left ventricle. (c) aortic valve. (d) and (k) compliance. (e) and (l) resistance. (f) and (m) systemic and pulmonary circulation reservoir. (g) tricuspid valve. (i) right ventricle. (j) pulmonary artery valve. (n) mitral valve

Figure S3

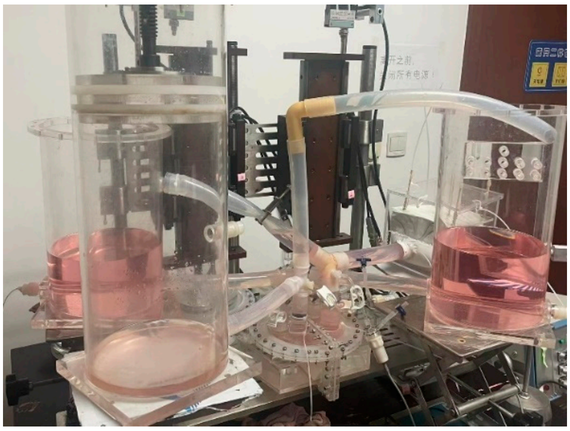

Figure S3 Physical picture of the MCL platform

Figure S4

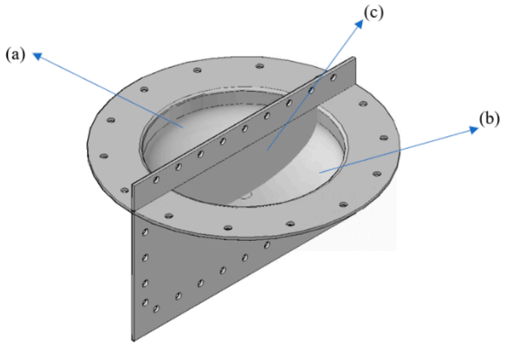

Figure S4 Ventricle Model. (a) and (b) left ventricle and right ventricle. (c) ventricular septum. (Holes at the edges are used for fixation)

Figure S5

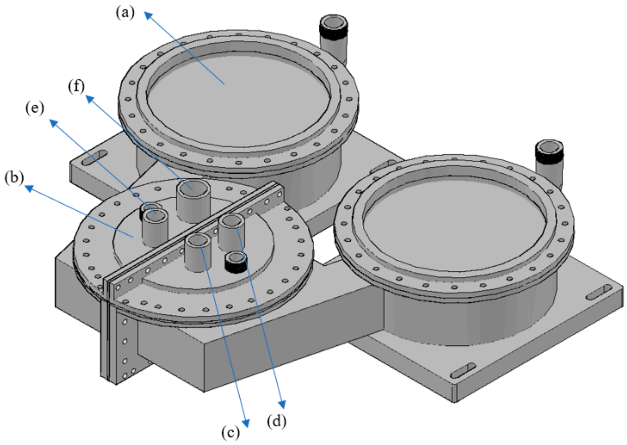

Figure S5 Drive and Fixed Part. (a) flexible diaphragm on which the motor acts directly. (b) Fixed position of the ventricular model. (c), (d), (e), (f) fixed position of pulmonary artery valve, tricuspid valve, aortic valve, mitral valve. (The other threaded channels are inlet and outlet or position of pressure measurement)

Figure S6

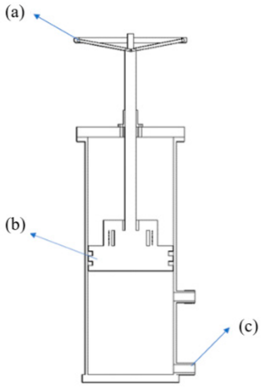

Figure S6 Compliance chamber of systemic circulation. (a) handwheel. (b) seal piston. (c) import and export of blood.

Figure S7

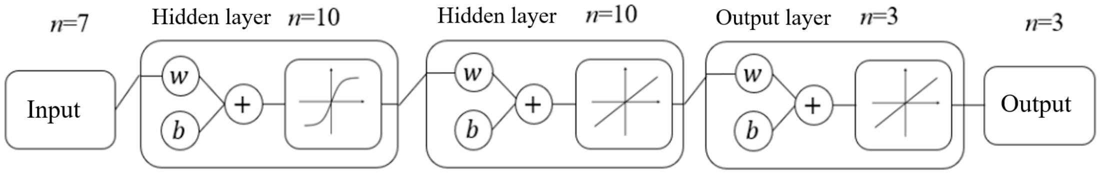

Figure S7 Structure diagram of Backward propagation network

Figure S8

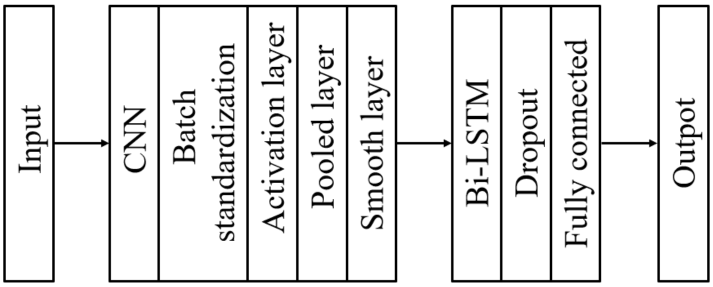

Figure S8 Structure diagram of deep learning model
